# Biochemical and Anti-Triple Negative Metastatic Breast Tumor Cell Properties of Psammaplins

**DOI:** 10.3390/md16110442

**Published:** 2018-11-10

**Authors:** Yu-Dong Zhou, Jun Li, Lin Du, Fakhri Mahdi, Thuy P. Le, Wei-Lun Chen, Steven M. Swanson, Kounosuke Watabe, Dale G. Nagle

**Affiliations:** 1 Institute of Interdisciplinary Integrative Medical Research, Shanghai University of Traditional Chinese Medicine, 1200 Cailun Road, Pudong New District, Shanghai 201203, China; 2 Department of Chemistry and Biochemistry, University of Mississippi, Oxford, MS 38677-1848, USA; traceeyle@gmail.com; 3 Department of BioMolecular Sciences and Research Institute of Pharmaceutical Sciences, School of Pharmacy, University of Mississippi, Oxford, MS 38677-1848, USA; drlj666@163.com (J.L.); Lin.Du-1@ou.edu (L.D.); fmahdi@olemiss.edu (F.M.); 4 Department of Medicinal Chemistry and Pharmacognosy, College of Pharmacy, University of Illinois at Chicago, Chicago, IL 60612, USA; wlchen1003@gmail.com; 5 Division of Pharmaceutical Sciences, School of Pharmacy, University of Wisconsin-Madison, Madison, WI 53705, USA; steve.swanson@wisc.edu; 6 Department of Cancer Biology, Wake Forest University School of Medicine, Winston-Salem, NC 27157, USA; kwatabe@wakehealth.edu

**Keywords:** psammaplins, HDAC inhibitors, HIF, VEGFA, anti-metastatic, metastatic organotropism, bone metastases, triple-negative breast cancer, metastases-specific antitumor agents, 3D spheroid invasion

## Abstract

Breast tumors reprogram their cellular metabolism, nutrient uptake, and utilization-associated biochemical processes. These processes become further transformed as genetically predisposed metastatic breast tumor cells colonize specific organs. Breast tumor cells often metastasize to the brain, bone, lung and liver. Massagué and colleagues isolated organotropic subclones and established organ-specific gene signatures associated with lung-, bone-, and brain-specific metastatic triple-negative breast cancer (TNBC) MDA-MB-231 cells. Using these genetically characterized metastatic subclones specific to lung (LM4175), bone (BoM1833), and brain (BrM-2a), we evaluated marine natural products for the ability to differentially suppress metastatic breast cancer cells in a target organ-dependent manner. Psammaplin-based histone deacetylase (HDAC) inhibitors were found to differentially inhibit HDAC activity, induce activation of hypoxia-inducible factor-1 (HIF-1), and disrupt organotropic metastatic TNBC subclone growth. Further, psammaplins distinctly suppressed the outgrowth of BoM1833 tumor spheroids in 3D-culture systems. Similar results were observed with the prototypical HDAC inhibitor trichostatin A (TSA). These organotropic tumor cell-based studies suggest the potential application of HDAC inhibitors that may yield new directions for anti-metastatic breast tumor research and drug discovery.

## 1. Introduction

Prevention and improved therapies have produced a steady decline in cancer rates in developed countries [1]. In spite of this success, systemic metastasis-associated disease relapse accounts for over 90% of cancer mortality. The five-year survival rate is 27% among the 162,000 American women with metastatic breast cancer [2]. Targeted therapies have limited success in stalling cancer progression and improving overall survival. Even highly responsive tumors often develop resistance by acquiring new mutations or by activating complimentary signaling pathways within a few months of treatment. Currently, there is still no treatment option that effectively curbs the spread of cancers to vital organs [3,4]. Metastatic disease remains a major cancer treatment challenge that warrants a more specific drug discovery approach.

The century-old “seed and soil” hypothesis of cancer metastasis compares systemically distributed tumor cells to “seeds,” and selected organs colonized by disseminated tumor cells as “soil.” Metastasis-initiating tumor cells invade and intravasate into the lymphatic vasculature and/or blood vessels, survive the circulation, extravasate to distant target organs, adapt to the new environment, and progress from dormancy to outgrowth into secondary lesions [3,5]. The inherent complexity of metastatic disease and technological limitations have hindered our molecular level understanding of metastasis and, subsequently, the discovery of anti-metastatic agents. Treatment options for metastatic breast cancer include surgery, radiation, chemotherapy, hormone, and molecular-targeted therapies. Since the landmark approval of trastuzumab in 1998, targeted therapies that include monoclonal antibodies, tyrosine kinase inhibitors, and poly (ADP-ribose) polymerase (PARP) inhibitors have been approved for metastatic breast cancer [6]. There are hundreds of targeted therapy-based clinical studies conducted every year [7]. However, the majority of agents in Phase II and III clinical trials assess secondary indications for previously approved therapeutic agents. Although targeted therapies can improve overall survival, few options exist for metastatic cancer and curative outcomes are negligible.

Metastatic organotropism represents an innovative anti-metastatic target. As heterogeneous populations, tumor cells vary significantly in gene expression pattern, differentiation status, and tumor metabolism. Breast cancers can metastasize to multiple organs (i.e., lung, bone, brain, liver, etc.). Genetic alterations and tumor-microenvironment interactions affect both metastatic propensity and organ tropism [3,4,5,6]. Massagué and colleagues revolutionized the field of metastasis research by establishing the gene signatures associated with organotropic metastatic breast cancers [8,9,10,11,12,13,14,15,16]. The lack of readily available molecular targets makes human triple-negative breast cancers (TNBCs) especially difficult to treat with any of the commonly used molecular-targeted antitumor drugs. Employing the widely studied TNBC cell line MDA-MB-231 as a model system, the Massagué group isolated organotropic subclones and identified signature gene expression profiles for lung-, bone-, and brain-specific breast cancer metastases [8,9,10,11,16]. The MDA-MB-231 metastatic subclones specific to lung (LM4175, LM), bone (BoM1833, BoM), and brain (BrM-2a, BrM) were obtained from the Massagué lab. With these recently established and genetically characterized in vitro models, we evaluated natural products for the ability to selectively suppress metastatic breast cancer cells in a target organ-dependent manner.

Marine invertebrate, algae, and terrestrial plant extracts (*n* = 880) and purified compounds (*n* = 3600) from the U.S. National Cancer Institute’s (NCI’s) Open Repository, and our purified natural product libraries, respectively, were evaluated for the ability to differentially suppress the growth of the organ-selective triple-negative metastatic subclonal lines, relative to their effects on less invasive T47D breast tumor cells, parent MDA-MB-231 cells, and other organ-selective MBA-MB-231 subtypes. The lipophilic extract of the marine sponge *Dendrilla lacunosa* Hentschel (Darwinellidae) and a set of histone deacetylase (HDAC) inhibitors known as psammaplins (isolated from the *D. lacunosa* extract) exhibited differential growth inhibitory activity against the MDA-MB-231-derived organotropic subclones [8,9,10,11,16]. Psammaplins were discovered by Crews and coworkers from *Pseudoceratina purpurea* and other sponges [17]. In general, HDAC inhibitors are believed to exert antitumor activity primarily through the epigenetic regulation of HDAC subtype-specific target gene expression [18,19]. This study examined these disulfide-bridged and oxime-substituted sponge metabolite psammaplins for their effects on MDA-MB-231 organotropic metastatic subclone proliferation/viability, HDAC activity, and the ability to regulate the expression of hypoxia-inducible factor 1 (HIF-1) target genes in vitro.

## 2. Results

### 2.1. Psammaplins Exhibit Concentration-Dependent Biphasic Effects on HIF-1 Activity

**Figure 1:**
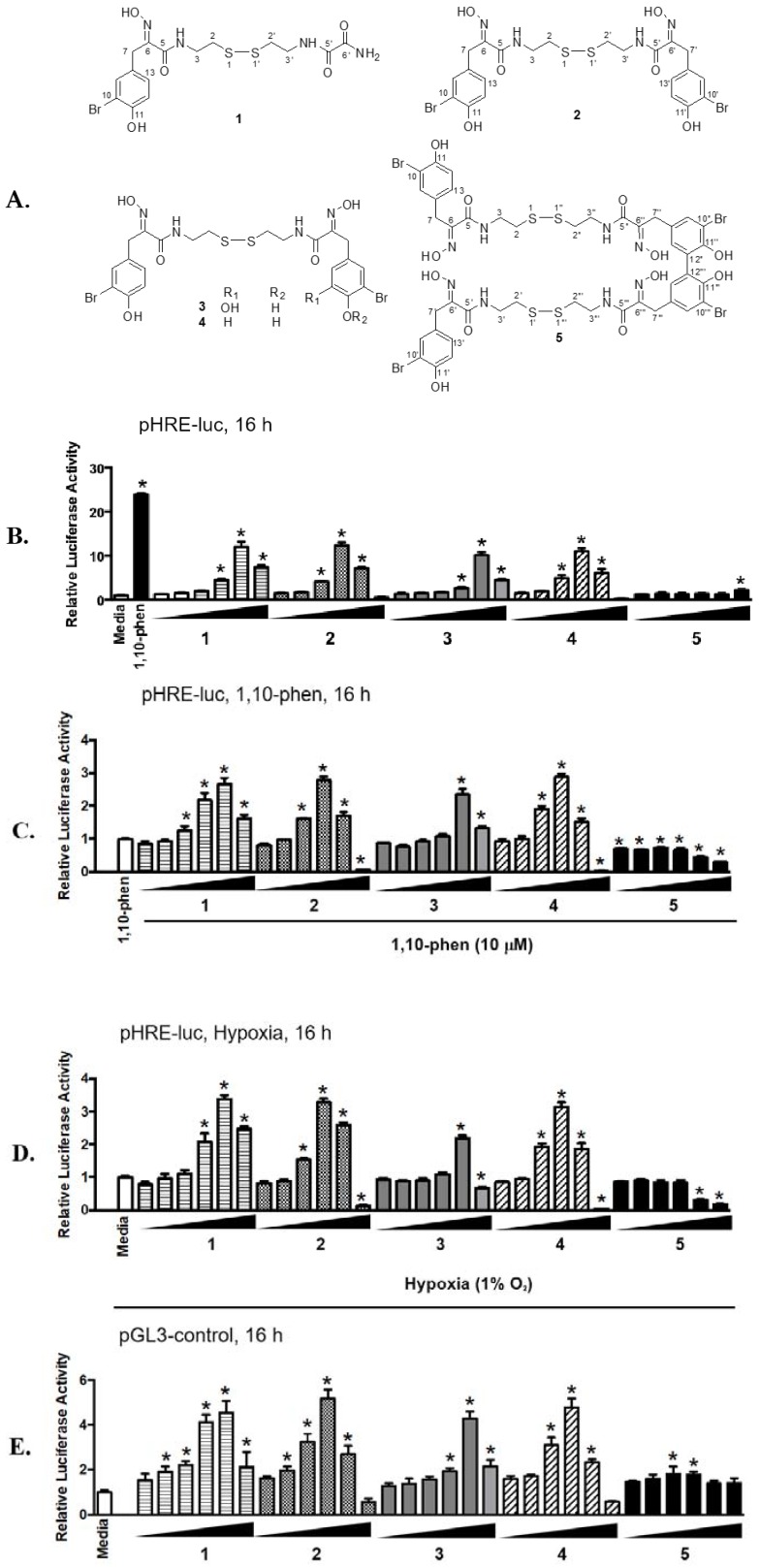
Concentration-dependent biphasic effects of **1**–**4** on HIF-1 activation. (**A**) Structures of psammaplins isolated from *Dendrilla lacunosa*. (**B**) Concentration-response results of **1**–**5** in T47D cells transfected with pHRE-luc for HIF-1 activity. Test compounds were added at the increasing concentrations of 0.1, 0.3, 1, 3, 10, and 30 µM, as specified. The positive control 1,10-phenanthroline (1,10-phen) was used at 10 µM. Data shown are average ± standard deviation (*n* = 3). (**C**) Similar to described in (**B**) except that the pHRE-luc transfected T47D cells were exposed to test compounds in the presence of 10 µM 1,10-phen, and the data were normalized to the positive control (1,10-phen). (**D**) Similar to described in (**C**) except that hypoxic exposure (1% O_2_: 5% CO_2_: 94% N_2_, 16 h) was applied in place of 1,10-phen. (**E**) As described in (**B**) except that T47D cells were transfected with the pGL3-control construct. An asterisk “*” indicates *p* < 0.05 when compared to the controls (“Media” for B, D, and E; and “1,10-phen” for C).

Cellular adaptation to hypoxia (low oxygen tension) is primarily mediated via the transcription factor hypoxia-inducible factor-1 (HIF-1), that regulates oxygen homeostasis by activating the expression of genes that increase oxygen availability and those that decrease oxygen consumption [20]. While HIF-1 is expressed ubiquitously, the human breast cancer T47D cell line displayed a robust response to hypoxia by activating HIF-1 and was used as an in vitro model to monitor HIF-1 activity. In a T47D cell-based reporter assay [21,22,23], a lipid extract sample of the sponge *Dendrilla lacunosa* activated HIF-1 by 3.56-fold (NIH collection No. C025691, 10 μg mL^−1^). Bioassay-guided fractionation of the extract sample (2.6 g) and chemical structure elucidation afforded five known compounds psammaplin E (**1**), (*E*,*Z*)-psammaplin A (**2**), (*E*,*E*)-psammaplin K (**3**), (*E*,*E*)-psammaplin A (**4**), and bisaprasin (**5**). The structures are shown in Figure 1A. To determine the effects of **1**–**5** on HIF-1 activity, concentration-response studies were performed in a T47D cell-based reporter assay (Figure 1B). An iron chelator (1,10-phenanthroline, 10 μM) was included as a positive control. Compounds **1**–**4** activated HIF-1 in a biphasic manner. The highest level of activation was observed at the concentrations of 3 μM for **2** and **4** [(12.32 ± 0.70)-fold and (11.01 ± 0.71)-fold, respectively, *n* = 3] and 10 μM for **1** and **3** [(12.01 ± 1.12)-fold and (10.15 ± 0.66)-fold, respectively, *n* = 3]. Compound **5** displayed weak HIF-1 activation at 30 μM [(2.17 ± 0.13)-fold, *n* = 3]. Hypoxia (1% O_2_) and chemical hypoxia (iron chelators or transition metals) represent two common stimuli that activate HIF-1 [24,25,26]. Further studies were performed to determine the effects of **1**–**5** on HIF-1 activity in the presence of other stimuli (1,10-phenanthroline, Figure 1C; hypoxia, Figure 1D). While **1**–**4** acted synergistically with 1,10-phenanthroline and hypoxia to activate HIF-1, a biphasic pattern of activation similar to that in the absence of stimulus (Figure 1A) was observed. In contrast, **5** inhibited HIF-1 activation at higher concentrations. Previous studies reported that psammaplins inhibit histone deacetylase (HDAC) enzymes [17,18]. To determine if HDAC inhibition non-specifically activates HIF-1, concentration-response studies were conducted in T47D cells transfected with the pGL3-control plasmid. As shown in Figure 1E, **1**–**4** enhanced luciferase activity in T47D cells transfected with the control plasmid. However, the activation of HIF-1 was significantly more pronounced than that of the pGL3-control (e.g., normalized ratio of pHRE-luc/pGL3-control at 2.64-fold for **1** at 10 μM, 2.38-fold for **2** at 3 μM, 2.38-fold for **3** at 10 μM, and 2.30-fold for **4** at 3 μM). These results suggest that **1**–**4** activated HIF-1 with specificity. At higher concentrations, the active compounds inhibited luciferase expression from both the pHRE-luc and the pGL3-control constructs. One possible scenario is that these compounds incur significant amount of cellular stress at higher concentrations, leading to the inhibition of gene expression in general.

### 2.2. Differential HDAC Inhibition by Psammaplin Analogues

The effects of **1**–**5** on HDAC activity were determined in a human melanoma MDA-MB-435 cell-based assay. Test compounds were added at specified concentrations to exponentially grown cells plated in 96-well plates. After 30 min incubation, the HDAC activity was determined using a commercial kit (HDAC-Glo™, Promega, Madison, WI, USA) and normalized to that of the dimethyl sulfoxide (DMSO) solvent control. The protypical HDAC inhibitors trichostatin A (TSA, 1 nM) and suberanilohydroxamic acid (SAHA, vorinostat, Zolinza^TM^, 100 nM) were included as positive controls. In the MDA-MB-435 cell-based assay, TSA and SAHA inhibited HDAC by 52 ± 8% and 60 ± 5%, respectively (average ± SE, *n* = 6). The IC_50_ values for **1**–**5** to inhibit HDAC are summarized in Table 1. The most potent compound (i.e., **2**) inhibited HDAC with an IC_50_ of 0.019 μM, while the least active (i.e., **5**) had an IC_50_ of 0.948 μM. The HDAC inhibitory activities of these compounds mirrored those observed in the T47D cell-based reporter assay (**2** is the most potent compound and was **5** the least potent).

### 2.3. Effects of Psammaplin A on HIF-1 Target Gene Expression

**Figure 2:**
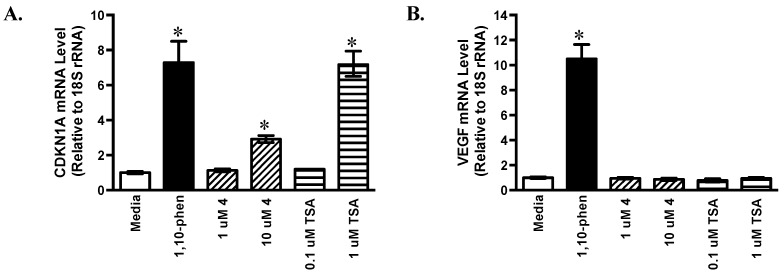

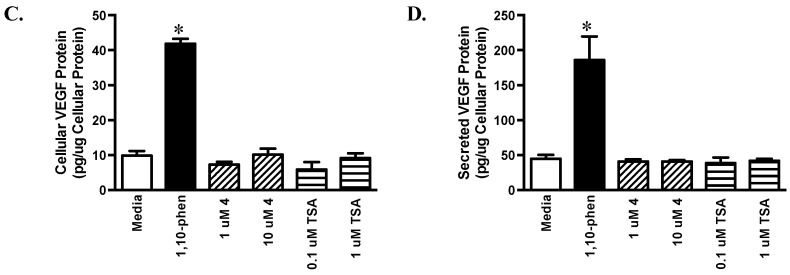
**Effects of 4 and trichostatin A (TSA) on HIF-1 target gene expression.** T47D cells were exposed to **4** and TSA at the specified concentrations for 16 h. The compound 1,10-phen (10 µM) was included as a positive control. The levels of cyclin dependent kinase inhibitor 1A (CDKN1A) (**A**) and vascular endothelial growth factor (VEGF) (**B**) mRNA following treatments were determined by quantitative real time RT-PCR. Relative levels of target gene mRNA normalized to an internal control (18S rRNA) are shown as average ± standard deviation (*n* = 3, one representative experiment). The levels of cellular (**C**) and secreted VEGF protein (**D**) were determined by ELISA and normalized to the amount of cellular proteins. Data shown are average + standard deviation (*n* = 3). An asterisk “*” indicates *p* < 0.05 when compared to the media control.

Around 100 genes have been identified as HIF-1 target genes that encode proteins involved in various aspects of cellular physiology [27]. While most of these genes are regulated in a cell type-specific manner, some are induced upon HIF-1 activation in most cell types [28]. Based on availability and potency, compound **4** (a stereoisomer of **2**) was selected for follow-up studies. The effects of **4** on the expression of HIF-1 target genes cyclin dependent kinase inhibitor 1A (*CDKN1A*) and vascular endothelial growth factor A (*VEGFA*) were examined by quantitative real time RT-PCR (Figure 2). The HIF-1 activator 1,10-phen (10 μM) and the pan-HDAC inhibitor TSA (0.1 and 1 μM, respectively) were included as positive controls. In T47D cells, **4** and TSA each increased the levels of CDKN1A mRNA in a concentration-dependent manner (2.9-fold for **4** at 10 μM and 7.2-fold for TSA at 1 μM, Figure 2A). In contrast, neither **4** nor TSA exerted greater than 20% effect on the levels of VEGF mRNA (Figure 2B). The gene VEGF encodes vascular endothelial growth factor A (a potent angiogenic factor) and agents that inhibit VEGF are in clinical use for cancer [29,30]. The expression of cellular and secreted VEGF proteins was examined in T47D cells by ELISA assay. As anticipated, the positive control 1,10-phen induced VEGF expression at the levels of mRNA (Figure 2B), cellular protein (Figure 2C), and secreted protein (Figure 2D). None of the HDAC inhibitors examined (**4** and TSA) increased VEGF protein levels at the concentrations tested (Figure 2C,D).

### 2.4. Psammaplins Suppress Cell Proliferation/Viability in a Cell Line-Dependent Manner

**Figure 3:**
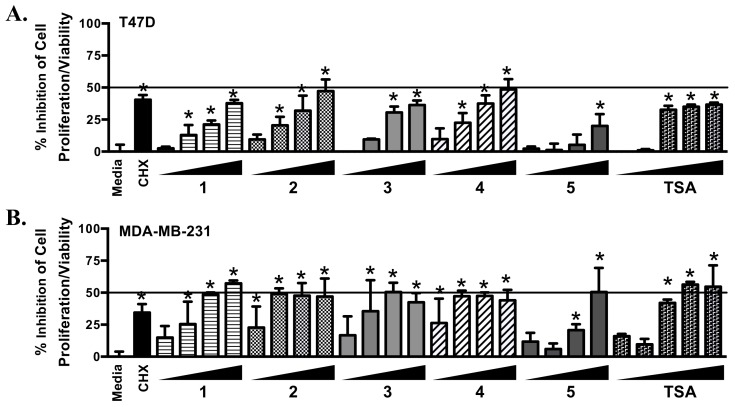

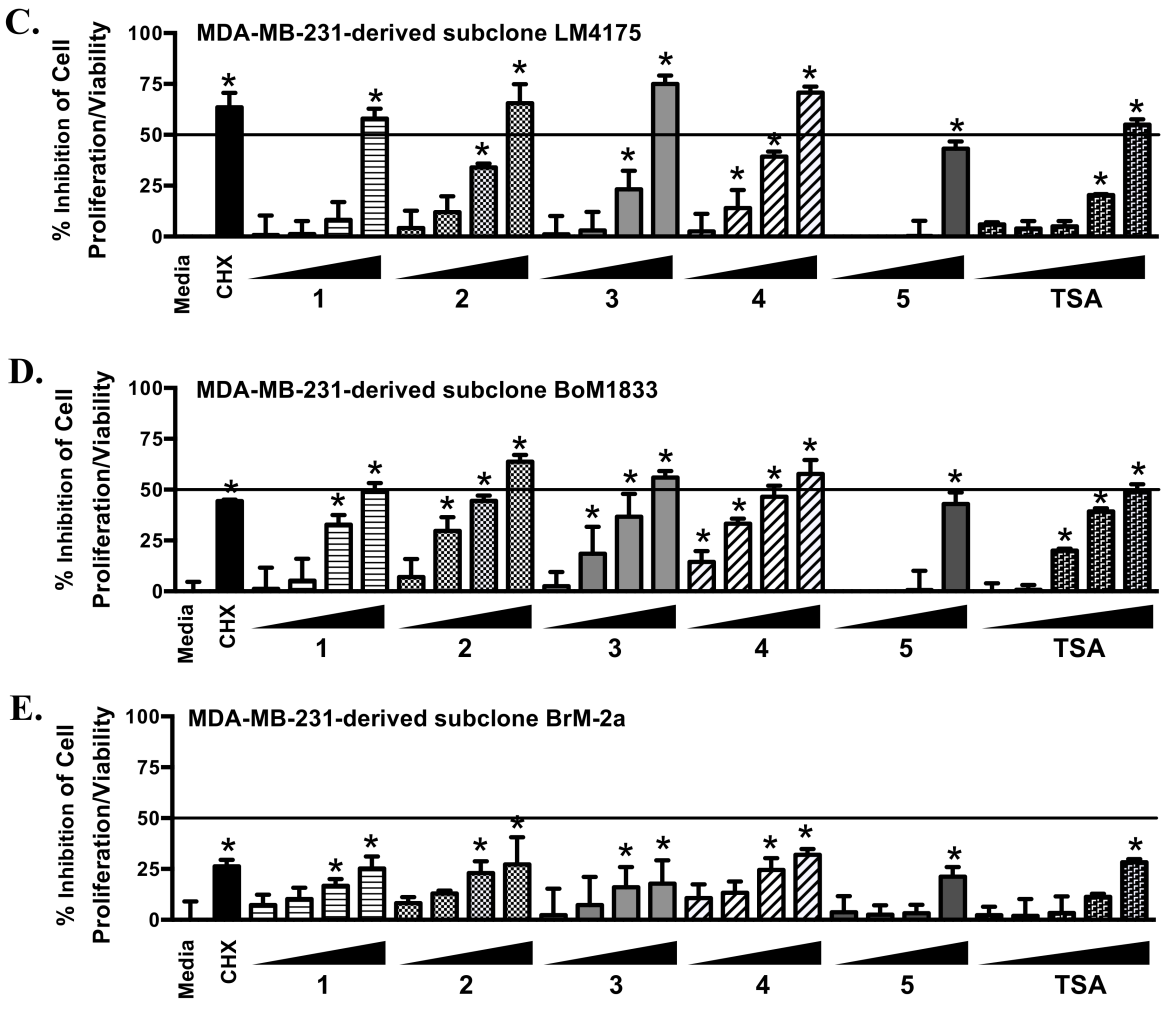
**Concentration-response results of 1–5 and TSA on cell proliferation/viability**. T47D (**A**), MDA-MB-231 (**B**), LM (**C**), BoM (**D**), and BrM (**E**) cells were exposed to **1**–**5** at the concentrations of 1, 3, 10, and 30 µM, TSA at 0.01, 0.03, 0.1, 0.3, and 1 µM, and CHX at 10 µM. After 48 h, cell viability was determined and presented as “% Inhibition” of the media control. Data shown are average + standard deviation, pooled from two experiments each performed in duplicate. An asterisk “*“ indicates *p* < 0.05 when compared to the media control.

In the T47D cell-based reporter assay, psammaplins regulated HIF-1 activity in a biphasic manner (Figure 1). To discern if cytostatic/cytotoxic effects contributed to the drop in HIF-1 activity at higher concentrations, the effects of psammaplins on cell proliferation/viability were examined in a panel of established human breast cancer cell lines. The protein synthesis inhibitor cycloheximide (CHX, 10 μM) was used as a positive control and the pan-HDAC inhibitor TSA was included for comparison. Following 48 h of compound treatment, all compounds affected cell proliferation/viability to a certain extent. Among the psammaplins, the potency rank of **2** and **4** > **3** > **1** > **5** mirrored that observed in the HDAC assay (Table 1). Greater inhibitory activity was observed in the TNBC MDA-MB-231, MDA-MB-231-derived bone metastatic BoM1833 (BoM) and lung metastatic LM4175 (LM) subclones, and the estrogen-dependent T47D cells, in comparison to the MDA-MB-231-derived brain metastatic subclone BrM-2a (BrM) (Figure 3). Similar cell line-dependent inhibitory activity was observed with TSA (Figure 3).

**Figure 4:**
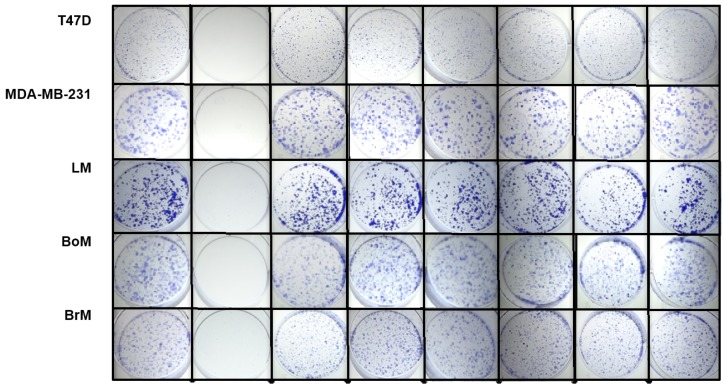
**Effects of 1–5 and TSA (trichostatin A) on colony formation.** Cells plated at low density were exposed to compounds (24 h) at specified concentrations (1 µM for paclitaxel and TSA, and 10 µM for **1**–**5**). After a period of ten days, the cells were fixed and stained.

The effects of psammaplins on the colony-forming ability of single cells were assessed in a clonogenic assay. Cells seeded at low density were exposed to test compounds at the specified concentrations for 24 h. The conditioned media were replaced with growth media and the colonies formed from surviving single cells in 10 days. While the cell lines differ in their colony-forming abilities, the positive control paclitaxel blocked colony formation in all cell lines (Figure 4). Less pronounced colony-suppressing activity was observed with the HDAC inhibitors.

### 2.5. Psammaplin A and TSA Inhibit Tumor Cell Invasion

**Figure 5:**
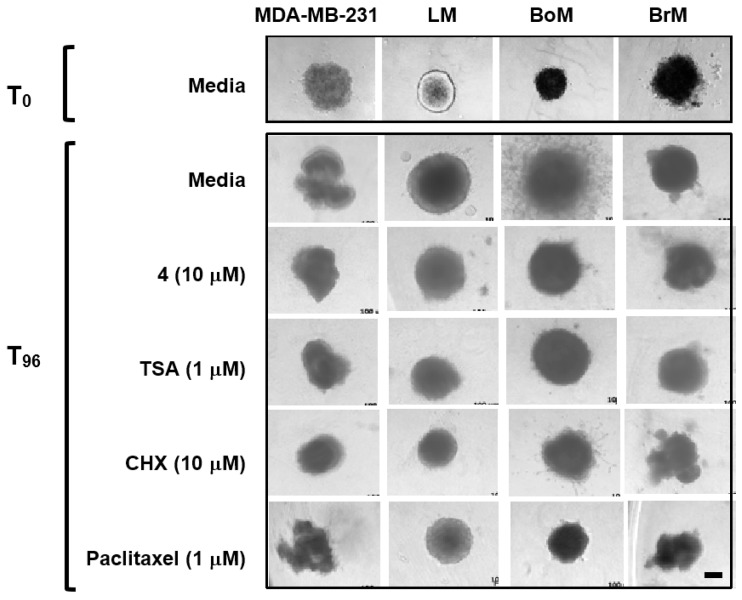
**Inhibition of bone metastatic BoM cell invasion.** Tumor cell spheroids formed in a special spheroid formation extracellular matrix (ECM) were embedded in an invasion matrix containing 10% fetal bovine serum (FBS) in the presence and absence of compounds at the specified concentrations. Four days later (T_96_), cell invasion was recorded microscopically (the bar at the bottom right represents 100 µm).

In order to form metastatic lesions, metastasis-initiating tumor cells must invade and intravasate into the lymphatic vasculature and/or blood vessels. Psammaplins were evaluated in a Cultrex^®^ 3-D cell invasion assay that monitors the invasion and migration of tumor cells grown as spheroids, which closely model in vivo pathophysiological conditions. The bone metastatic BoM subclone displayed the most aggressive behavior (a network of extensive projections from the spheroid, T_96_, Media, Figure 5). Compound **4** and TSA each inhibited the invasion of BoM spheroids into the extracellular matrix (ECM), similar to those observed in the presence of the positive controls, paclitaxel and CHX (Figure 5). Further, a more pronounced decrease in the size of the spheroids was observed in the presence of paclitaxel and CHX, in comparison to the HDAC inhibitors.

## 3. Discussion

The overwhelming majority of all targeted therapies approved for the treatment of breast cancer specifically target either estrogen or progesterone hormone receptors (e.g., tamoxifen, anastrozole, letrozole) or target tumors that overexpress the proto-oncogenic receptor tyrosine-protein kinase known as human epidermal growth factor receptor 2 (HER2) (e.g., trastuzumab, pertuzumab) [31]. Human TNBCs represent a major treatment challenge because of the lack of estrogen receptor (ER), progesterone receptor (PR), and HER2. Genetic studies identified at least half a dozen distinct TNBC subtypes with unique gene expression profiles that appear to respond differentially to NVP-BEZ235 (PI3K/mTOR inhibitor), dasatinib (abl/src inhibitor), and the androgen receptor antagonist bicalutamide [32]. Mooberry and coworkers recently demonstrated that certain DNA damaging agents, such as the *Tolypocladium* sp. fungi hybrid polyketide-shikimate-nonribosomal peptide synthetase metabolite, maximiscin, exhibits a differential pattern of triple-negative breast tumor cell line selectivity [33]. It is clear that small molecules selectively target various subtypes of primarily tumor biopsy-derived TNBCs can be identified through targeted screening efforts.

To discover potential new agents that differentially inhibit patient-derived TNBC metastases or specifically target organotropic metastatic TNBCs, we initiated a screening campaign using the TNBC MDA-MB-231 derived organotropic clones as in vitro models. Our initial screening efforts indicate that natural product-based HDAC inhibitors (i.e., psammaplins and trichostatin A) differentially suppress the proliferation and three-dimensional invasive growth potential of TNBC cells that are oncogenically predisposed to colonize specific organs. These psammaplins (**1**–**4**) were isolated from the sponge *Dendrilla lacunosa* for their HIF inducing activity. The most potent two compounds (*E*,*Z*)-psammaplin A (**2**) and (*E*,*E*)-psammaplin A (**4**) are stereo isomers, while the structurally related compounds psammaplin E (**1**) and (*E*,*E*)-psammaplin K (**3**) are less active. In T47D cell-based reporter assays, psammaplins displayed biphasic effects (induction at lower concentrations and inhibition at higher concentrations). This is not surprising because hypoxia and chemical hypoxia impose cellular stress. The extent of damage caused by severe hypoxia or high concentrations of chemicals that activate hypoxic signaling may induce senescence or cell death. Either of these conditions will result in decreased reporter gene expression. In comparison to the common HIF stimuli (e.g., hypoxia, iron chelator, etc.), (*E*,*E*)-psammaplin A (**4**) and the HDAC inhibitor standard TSA are unique in the aspect that they induced the expression of cyclin dependent kinase inhibitor 1A (*CDKN1A*), but not the expression of vascular endothelial growth factor A (*VEGFA*). While both are HIF-1 target genes, *CDKN1A* stalls cell cycle progression and *VEGFA* promotes tumor angiogenesis. Further study is required to resolve the mechanisms behind the differential effects exerted by HDAC inhibitors on HIF-1 target gene expression. Clinically, the natural product HDAC inhibitor romidepsin (Istodax^TM^) and SAHA (vorinostat, Zolinza^TM^) are used for the treatment of cutaneous T-cell lymphoma [34,35]. However, HDAC inhibitors have not found general utility in the treatment of genetically diverse primary solid tumors.

Recent studies suggest that psammaplins induce Sirtuin 1-dependent autophagic cell death in human breast tumor cell lines and xenografts [36,37]. Psammaplin A decreased SIRT1 enzyme expression and activity in breast tumor cells, increased the acetylation of the SIRT1 target p53, and produced an overall increase in autophagy-related protein expression, including that of the p53-induced protein, DRAM (damage-regulated autophagy modulator). It is possible that psammaplins stall tumor progression by inducing the expression of proliferation inhibiting and cell death promoting genes, without stimulating the expression of survival genes (i.e., *VEGFA*). Among the three MDA-MB-231 derived organotropic clones examined, the slowest growing BrM subclone was least affected by psammaplins. The most aggressive BoM cells invaded extracellular matrix in a 3D spheroid invasion assay and (*E*,*E*)-psammaplin A (**4**) inhibited this invasion. Compound **4** and the standard HDAC inhibitor TSA were tested at the IC_50_ values determined in a 48 h proliferation/viability. Because the invasion assay was conducted over the course of 96 h, it is also possible that target proteins other than histones (e.g., tubulins) were affected by HDAC inhibitors. Increased acetylation of these non-histone target proteins may directly contribute to the blockade of cell invasion.

The concept of natural product-derived HDAC inhibitors as potential antimetastatic agents that target TNBCs is further supported by the work of Lu, Wang, and coworkers with garlic diallyl trisulfide that acts as a natural HDAC inhibitor [38]. Garlic diallyl trisulfide was found to suppress MDA-MB-231 metastatic potential in embryonic zebrafish, xenografts, and orthotopic tumor models, and inhibited MDA-MB-231 migration and angiogenesis in vitro. Although these are only the first efforts to identify natural product-derived agents with potential to selectively suppress organotropic metastatic TNBCs, they provide vital new prospects for the repurposing of clinically approved chemotherapeutic drugs and other natural product-based agents in the treatment or chemoprevention of organ-specific TNBC metastases.

## 4. Materials and Methods

### 4.1. General Experimental Procedures

Human breast tumor T47D and MDA-MB-231 cells were from ATCC (Manassas, VA, USA). The MDA-MB-231-derived subclones BoM1833 (BoM, bone metastatic), LM4175 (LM, lung metastatic), and BrM-2a (BrM, brain metastatic) were obtained from Dr. J. Massagué at Memorial Sloan Kettering Cancer Center, New York City, NY, USA. Cells were maintained in DMEM/F12 media with L-glutamine (Mediatech, Manassas, VA, USA), supplemented with 10% (v/v) fetal bovine serum (FBS, Hyclone, Logan, UT, USA), 50 units/mL penicillin and 50 µg/mL streptomycin (Gibco, Grand Island, NY, USA) at 37 °C in a humidified environment under 5% CO_2_:95% Air. Unless specified, all other chemicals were purchased from Sigma-Aldrich (St. Louis, MO, USA).

### 4.2. Sponge Material, Extract Preparation, and Bioassay-Guided Isolation

The sponge material was part of the NCI Open Repository Collection. *Dendrilla lacunosa* was collected by Don DeMaria (sub-contractor to the Coral Reef Research Foundation; NPID No. C025691) at a depth of 5−8 m off the coast of the Northern Territory of Australia. It was identified by Dr. Patricia R. Bergquist and a voucher specimen (collection number 0M9H2419) placed on file with the Department of Invertebrate Zoology, National Museum of Natural History, Smithsonian Institution, Washington, DC, USA. After freezing at −20 °C, the *D. lacunosa* sponge sample was ground in a meat grinder, extracted with water, the residual sample lyophilized and extracted with 50% MeOH in CH_2_Cl_2_ [39]. The solvents were later removed under vacuum and the extract sample stored at −20 °C (NCI repository, Frederick Cancer Research and Development Center, Frederick, MD, USA).

The *D. lacunosa* extract activated HIF-1 in a T47D cell-based reporter assay (3.56-fold at 10 μg mL^−1^). The *D. lacunosa* extract (2.6 g) was suspended in 50% MeOH in CH_2_Cl_2_, filtered to remove residue, and separated into eight fractions by Sephadex LH-20 column (eluted with CH_2_Cl_2_/MeOH, 50:50). The sixth fraction (HIF-1 activation by 2.97-fold, 1.0 μg mL^−1^, 285 mg) was separated by a semi-preparative HPLC [Luna 5 μm, C18(2) 100 Å, 250 × 10.0 mm, isocratic 63% MeOH in H_2_O, 4.0 mL min^−1^], to produce psammaplin E (**1**, 0.8 mg, 0.03% yield, *t*_R_ 6.1 min), (*E*,*Z*)-psammaplin A (**2**, 1.8 mg, 0.07% yield, *t*_R_ 10.3 min), (*E*,*E*)-psammaplin K (**3**, 20 mg, 0.76% yield, *t*_R_ 11.3 min) and (*E*,*E*)-psammaplin A (**4**, 155 mg, 5.92% yield, *t*_R_ 14.3 min). The MeOH eluate from HPLC column was purified by semi-preparative HPLC [Luna 5 μm, C18(2) 100 Å, 250 × 10.0 mm, isocratic 70% MeOH in 0.1% TFA/H_2_O, 4.0 mL min^−1^], to afford bisaprasin (**5**, 6.7 mg, 0.25% yield, *t*_R_ 18.2 min). The purified psammaplins were confirmed to be greater than 95% pure by ^1^H NMR.

### 4.3. Structural Data

**Psammaplin E** (**1**): oil, positive ion ESI-MS, *m*/*z* 479.0/481.0 [M + H]^+^, *m*/*z* 501.0/503.0 [M + Na]^+^; ^1^H NMR (CDCl_3_, 400 MHz): *δ* 7.37 (1H, d, *J* = 2.0 Hz, H-9), 7.08 (1H, dd, *J* = 8.0, 2.0 Hz, H-13), 6.77 (1H, d, *J* = 8.0 Hz, H-12), 3.80 (2H, s, H-7), 3.56 (4H, m, H-3,3′), 2.86 (4H, t, *J* = 6.8 Hz, H-2,2′). The structure of **1** was confirmed by comparison with previously published ^1^H-NMR data [17].

**(*E*,*Z*)-Psammaplin A** (**2**): gum, positive ion ESI-MS, *m*/*z* 663.0/665.0/667.0 [M + H]^+^, *m*/*z* 684.9/686.9/688.9 [M + Na]^+^; ^1^H NMR (CD_3_OD, 400 MHz): *δ* 7.37 (1H, d, *J* = 2.0 Hz, H-9), 7.32 (1H, d, *J* = 2.0 Hz, H-9′), 7.07 (1H, dd, *J* = 8.0, 2.0 Hz, H-13′), 7.02 (1H, dd, *J* = 8.0, 2.0 Hz, H-13), 6.79 (1H, d, *J* = 8.0 Hz, H-12), 6.77 (1H, d, *J* = 8.0 Hz, H-12′), 3.80 (2H, s, H-7), 3.59 (2H, s, H-7′), 3.52 (2H, t, *J* = 6.8 Hz, H-3), 3.50 (2H, t, *J* = 6.8 Hz, H-3′), 2.79 (2H, t, *J* = 7.0 Hz, H-2), 2.72 (2H, t, *J* = 7.0 Hz, H-2′); ^13^C NMR (CD_3_OD, 100 MHz): *δ* 164.5 (C-5′), 163.0 (C-5), 152.9 (C-6′), 152.4 (C-6), 151.7 (C-11) 151.4 (C-11′), 133.1 (C-9, 9′), 129.1 (C-13′), 129.0 (C-13), 128.9 (C-8), 128.7 (C-8′), 115.8 (C-12) 115.7 (C-12′), 109.4 (C-10), 109.1 (C-10′), 38.2 (C-3′), 37.9 (C-3), 37.0 (C-2, 2′), 36.8 (C-7′), 27.3 (C-7). The structure of **2** was confirmed by comparison with previously published ^1^H-NMR and ^13^C-NMR data [40].

**(*E*,*E*)-psammaplin K** (**3**): gum, positive ion ESI-MS, *m*/*z* 679.0/681.0/683.0 [M + H]^+^, *m*/*z* 700.9/702.9/704.9 [M + Na]^+^; ^1^H NMR (CDCl_3_, 400 MHz): *δ* 7.36 (1H, br s, H-9), 7.06 (1H, d, *J* = 7.2 Hz, H-13), 6.85 (1H, s, H-9′), 6.75 (1H, d, *J* = 8.0 Hz, H-12), 6.71 (1H, s, H-13′), 3.79 (2H, s, H-7′), 3.73 (2H, s, H-7), 3.51 (4H, t, *J* = 6.8 Hz, H-3, 3′), 2.80 (4H, t, *J* = 6.8 Hz, H-2, 2′); ^13^C NMR (CDCl_3_, 100 MHz): *δ* 164.5 (C-5, 5′), 152.3 (C-6), 151.7 (C-11, 6′), 145.7(C-12′), 141.1 (C-11′), 133.1(C-9), 129.2 (C-13), 129.0 (C-8, 8′), 123.4 (C-9′), 115.6 (C-12), 115.0 (C-13′), 109.1 (C-10, 10′), 38.2 (C-3, 3′), 37.1 (C-2, 2′), 27.5 (C-7′), 27.3 (C-7). The structure of **3** was confirmed by comparison with previously published ^1^H-NMR and ^13^C-NMR data [40].

**(*E*,*E*)-psammaplin A** (**4**): white powder, positive ion ESI-MS, *m*/*z* 663.0/665.0/667.0 [M + H]^+^, *m*/*z* 684.9/686.9/688.9 [M + Na]^+^; ^1^H NMR (CD_3_OD, 400 MHz): *δ* 7.36 (2H, d, *J* = 2.0 Hz, H-9, 9′), 7.06 (2H, dd, *J* = 8.4, 2.0 Hz, H-13, 13′), 6.75 (2H, d, *J* = 8.4 Hz, H-12, 12′), 3.79 (4H, s, H-7, 7′), 3.51 (4H, t, *J* = 6.8 Hz, H-3, 3′), 2.79 (4H, t, *J* = 6.8 Hz, H-2, 2′); ^13^C NMR (CD_3_OD, 100 MHz): *δ* 164.5 (C-5, 5′), 152.3 (C-6, 6′), 151.7 (C-11, 11′), 133.1 (C-9, 9′), 129.2 (C-13, 13′), 129.0 (C-8, 8′), 115.6 (C-12, 12′), 109.1 (C-10, 10′), 38.2 (C-3, 3′), 37.1 (C-2, 2′), 27.3 (C-7, 7′). The structure of **4** was confirmed by comparison with previously published ^1^H-NMR and ^13^C-NMR data [41].

**Bisaprasin** (**5**): gum, negative ion ESI-MS, *m*/*z* 660.9/661.9/662.9 [M − 2H]^2−^; ^1^H NMR (CD_3_OD, 400 MHz): *δ* 7.42 (2H, br s, H-9″,9‴), 7.37 (2H, br s, H-9, 9′), 7.06 (4H, m, H-13, 13′, 13″, 13‴), 6.77 (1H, m, H-12, 12′), 3.85 (4H, s, H-7″,7″), 3.79 (4H, s, H-7, 7′), 3.52 (8H, m, H-3, 3′, 3″, 3‴), 2.79 (8H, m, H-2, 2′, 2″, 2‴); ^13^C NMR (CD_3_OD, 100 MHz): *δ* 164.5 (C-5, 5′, 5″, 5‴), 152.3 (C-11, 11′), 151.7 (C-6,6′), 151.6 (C-6″,6‴), 149.2 (C-11″, 11‴), 133.1 (C-9, 9′), 132.6 (C-9″, 9‴), 131.2 (C-13, 13′), 129.7 (C-8), 129.2 (C-8′), 129.0 (C-13″, 13‴), 127.2 (C-8″, 8‴), 115.7 (C-12, 12′), 111.6 (C-12″, 12‴), 109.1 (C-10, 10′, 10″, 10‴), 38.2 (C-3, 3′, 3″, 3‴), 37.1 (C-2, 2′, 2″, 2‴), 27.4 (C-7″, 7‴), 27.3 (C-7, 7′). The structure of **5** was confirmed by comparison with previously published ^1^H-NMR and ^13^C-NMR data [40,41,42].

### 4.4. T47D Cell-Based Reporter Assay

To monitor HIF-1 activity, T47D cells were transfected with the pHRE3-TK-Luc construct and the cell-based luciferase reporter assay was performed as described [20]. Cells were exposed to test compounds in the absence and presence of 1,10-phenanthroline (10 μM) or hypoxic conditions (1% O_2_: 5% CO_2_: 94% N_2_) for 16 h. The cells were lysed and luciferase activity determined with a commercial kit (Promega, Madison, WI, USA). For the cell-based control reporter assay, T47D cells were transfected with the pGL3-control construct (Promega, Madison, WI, USA), exposed to test compounds for 16 h, and the luciferase reporter assay performed as described [21].

### 4.5. MDA-MB-435 Cell-Based HDAC Assay

Human melanoma MDA-MB-435 cells (ATCC, Manassas, VA, USA) were maintained in RPMI 1640 medium supplemented with 10% FBS, 100 units/mL penicillin, and 100 μg/mL streptomycin. Exponentially grown cells were seeded at the density of 5000 cells/well into 96-well plates (Corning, Corning, NY, USA) and incubated overnight. Compounds dissolved in DMSO were added to achieve the specified final concentrations (total volume: 100 μL, DMSO: 0.5%). The incubation continued for 30 min at 37 °C and the HDAC activity determined using a commercial luminescent assay (HDAC-Glo™, Promega, Madison, WI, USA). The HDAC inhibitors trichostatin A (TSA, 1 nM) and SAHA (100 nM) were used as positive controls and the data presented as percentage inhibition of the solvent control.

### 4.6. Quantitative Real-Time RT-PCR and ELISA Assay

The effects of test samples on HIF-1 target gene expression were assessed in T47D cells. To determine the levels of CDKN1A and VEGF mRNA, quantitative real-time RT-PCR was performed as described [21,43]. To determine the levels of cellular and secreted VEGF proteins, T47D cells were exposed to compounds as described [44], the levels of VEGF proteins in the conditioned medium and cell lysate samples determined by ELISA [21], the amount of proteins in the cell lysate samples quantified using a micro BCA assay kit (Thermo Fisher Scientific, Rockford, IL, USA), and the level of VEGF proteins normalized to that of cellular proteins.

### 4.7. Cell Proliferation/Viability and Clonogenic Survival Assays

The cell proliferation/viability assay (48 h exposure) was performed as described, using the sulforhodamine B method [22]. The data are presented as ‘% Inhibition’ of the media control.

For the clonogenic assay, exponentially grown cells were seeded at the density of 1000 cells/well into 6-well plates (Cellstar^®^, Greiner Bio-One GmbH, Kremsmünster, Austria) and incubated at 37 °C for 4 h to allow the cells to adhere. Compound addition was similar as above. After 24 h, the compound-containing conditioned media were replaced with fresh medium containing FBS (10%) and antibiotics. The incubation continued for another 10 days with a change of fresh medium every 5 days, the cells were fixed with methanol and stained with crystal violet (1 mg/mL in 20% ethanol), and the images were acquired with a Kodak digital camera.

### 4.8. 3D Cell Invasion Assay

The 3D spheroid cell invasion assay was performed using a commercial kit, following the manufacturer’s instructions (Cultrex^®^ 3D Spheroid Cell Invasion Assay, Trevigen, Gaithersburg, MD, USA). Briefly, exponentially grown MDA-MB-231, LM, BoM, and BrM cells were trypsinized, collected, and resuspended in serum-free DMEM/F12 media. For spheroid formation, 3000 cells were added in a volume of 50 µL serum-free DMEM/F12 media with 1× spheroid formation solution/well into a pre-cooled 96-well plate. The plate was centrifuged at 100× *g* for 5 min at 4 °C, incubated at 37 °C for 24 h, and the spheroids imaged using an Axiovert 40 CFL microscope (Zeiss, Oberkochen, Germany). The plate was placed on ice, the invasion mix added in a volume of 50 µL/well, centrifuged at 350× *g* for 5 min at 4 °C, and incubated at 37 °C for 1 h. Test compounds and controls were diluted to two times the final concentrations in DMEM/F12 media supplemented with FBS (10%) and antibiotics, and added in a volume of 100 µL/well. The incubation continued for another 4 days at 37 °C and the cells/spheroids imaged (T_96_).

### 4.9. Statistical Analysis

GraphPad Prism 6 was applied to analyzed data. Data were compared by one-way ANOVA followed by Bonferroni post-hoc analyses. Differences were considered statistically significant when *p* < 0.05.

## Figures and Tables

**Table 1:** **IC_50_ values of 1–5 in an MDA-MB-435 cell-based histone deacetylase (HDAC) assay**.

Compound	IC_50_ (µM)	95% CI (µM) ^1^
**1**	0.257	0.157–0.420
**2**	0.019	0.012–0.028
**3**	0.038	0.024–0.061
**4**	0.037	0.025–0.055
**5**	0.948	0.586–1.532

^1^ Data from two independent experiments (*n* = 6) were pooled to calculate IC_50_. The 95% confidence interval (95% CI). IC_50_ values are also provided. Inhibition by protypical HDAC inhibitor controls: trichostatin A (TSA, 1 nM) 52 ± 8%; vorinostat (SAHA, 100 nM) 60 ± 5%.
